# DNA molecular combing-based replication fork directionality profiling

**DOI:** 10.1093/nar/gkab219

**Published:** 2021-04-09

**Authors:** Marion Blin, Laurent Lacroix, Nataliya Petryk, Yan Jaszczyszyn, Chun-Long Chen, Olivier Hyrien, Benoît Le Tallec

**Affiliations:** Département de Gastro-entérologie, pôle MAD, Assistance Publique des Hôpitaux de Marseille, Centre Hospitalier Universitaire de Marseille, Marseille, France; Institut de Biologie de l’Ecole Normale Supérieure (IBENS), Ecole Normale Supérieure, CNRS, INSERM, Université PSL, 46 rue d’Ulm, F-75005 Paris, France; Institut de Biologie de l’Ecole Normale Supérieure (IBENS), Ecole Normale Supérieure, CNRS, INSERM, Université PSL, 46 rue d’Ulm, F-75005 Paris, France; Université Paris-Saclay, CEA, CNRS, Institute for Integrative Biology of the Cell (I2BC), F-91198 Gif-sur-Yvette, France; Université Paris-Saclay, CEA, CNRS, Institute for Integrative Biology of the Cell (I2BC), F-91198 Gif-sur-Yvette, France; Institut Curie, Université PSL, Sorbonne Université, CNRS UMR3244, F-75005 Paris, France; Institut de Biologie de l’Ecole Normale Supérieure (IBENS), Ecole Normale Supérieure, CNRS, INSERM, Université PSL, 46 rue d’Ulm, F-75005 Paris, France; Institut de Biologie de l’Ecole Normale Supérieure (IBENS), Ecole Normale Supérieure, CNRS, INSERM, Université PSL, 46 rue d’Ulm, F-75005 Paris, France

## Abstract

The replication strategy of metazoan genomes is still unclear, mainly because definitive maps of replication origins are missing. High-throughput methods are based on population average and thus may exclusively identify efficient initiation sites, whereas inefficient origins go undetected. Single-molecule analyses of specific loci can detect both common and rare initiation events along the targeted regions. However, these usually concentrate on positioning individual events, which only gives an overview of the replication dynamics. Here, we computed the replication fork directionality (RFD) profiles of two large genes in different transcriptional states in chicken DT40 cells, namely untranscribed and transcribed *DMD* and *CCSER1* expressed at WT levels or overexpressed, by aggregating hundreds of oriented replication tracks detected on individual DNA fibres stretched by molecular combing. These profiles reconstituted RFD domains composed of zones of initiation flanking a zone of termination originally observed in mammalian genomes and were highly consistent with independent population-averaging profiles generated by Okazaki fragment sequencing. Importantly, we demonstrate that inefficient origins do not appear as detectable RFD shifts, explaining why dispersed initiation has remained invisible to population-based assays. Our method can both generate quantitative profiles and identify discrete events, thereby constituting a comprehensive approach to study metazoan genome replication.

## INTRODUCTION

DNA replication is under active investigation in higher eukaryotes, but maps of replication origins unanimously agreed upon are still lacking. Over the past two decades, analyses using high-throughput, population-averaging replication profiling techniques have led to conflicting data, pointing out either highly specific sites of replication initiation associated with sequence elements or broad initiation zones with no particular genetic determinants (1–3). Genome-wide identification of the location of replication initiation by population-based approaches is challenging given the remarkable flexibility of metazoan origins. Only efficient initiation at specific sites or zones may escape background levels using ensemble-averaging origin mapping assays, whether they rely on the analysis of short nascent strands (for instance (4–10)), Okazaki fragments (11–19), trapped replication bubbles (20,21), chromatin immunoprecipitation of licensing factors (22–26) or alternative strategies (13,27–30), whereas inefficient origins remain hidden.

Single-molecule (SM) methods are capable of revealing rare initiation events. DNA fibre stretching by DNA spreading or molecular combing, combined with the immunodetection of distinct thymidine analogs sequentially incorporated in cells grown *in vitro*, allows direct visualisation of newly synthesized DNA along individual molecules and discrimination between elongating forks, initiation and termination events (31–33). Moreover, together with the use of fluorescence *in situ* hybridization (FISH) probes, DNA fibre analysis can map replication signals within a specific locus (34). However, the throughput of classical SM techniques is drastically low and incompatible with genome-wide studies. Very recently, novel nanopore sequencing-based methods to study DNA replication at the SM level, namely DNAscent (35), FORK-seq (36) and Replipore sequencing (37), tipped SM analyses into the high-throughput era. Noticeably, both DNAscent and FORK-seq techniques successfully reproduced population-based replication profiles of the yeast *Saccharomyces cerevisiae* genome from the assembly of thousands of individual replication signals, while uncovering that, in addition to efficient initiation at known origins, a significant proportion (9–20%) of initiation takes place at dispersed, inefficient sites previously missed by population-based assays (35,36). This novel generation of SM techniques, together with the development of high-throughput optical mapping of DNA replication (38–41), will undoubtedly allow routine, genome-wide replication profiling in higher eukaryotes in the near future. Still, current nanopore-based methods are limited by the sequencing costs to achieve high coverage studies of metazoan genome replication, while optical replication mapping approaches are presently unable to measure fork velocity, a critical replication parameter. At the same time, standard SM analyses of specific loci in metazoans, which can theoretically provide a comprehensive analysis of the replication dynamics of the targeted locus, have been underexploited. These usually concentrate on positioning individual replication signals, particularly initiation and termination events (34,42–46), leaving aside the opportunity to assemble a reliable replication fork directionality (RFD) profile of the locus from the collection of a large number of oriented replication tracks, similar to what was done genome-wide in yeast by FORK-seq (36). RFD reveals the proportion of rightward- and leftward-replicated DNA and allows a quantitative analysis of replication initiation, progression and termination (11–19,47–51).

We have recently used DNA combing to demonstrate that transcriptional activity modulates origin distribution in the 616- and 996-kb-long *CCSER1* and *DMD* genes, respectively, in chicken DT40 cells, and proposed that transcription favours efficient initiation at the 5′ and 3′ ends of large genes at the expense of the gene body (46). Here, we reanalyzed these datasets and extracted the orientation of each replication track to compute the RFD profiles of inactive and active *DMD*, as well as those of *CCSER1* when it is transcribed at wild-type (WT) levels or overexpressed. In order to compare SM- and population-based approaches, we also generated the RFD profiles of *DMD* and *CCSER1* in WT cells using Okazaki fragment sequencing (OK-seq) (11,14). We show that DNA combing- and OK-seq-based RFD profiles are highly concordant, with DNA combing-based RFD successfully reproducing domains of replication composed of two zones of initiation flanking a zone of termination detected by OK-seq. Importantly, we demonstrate that a significant portion of initiation events mapped on single molecules are too diffuse to generate signals indicative of initiation on the RFD profiles, explaining why dispersed origin firing has largely been missed by population-based techniques. By combining the ability to build accurate replication profiles with the capacity to monitor cell-to-cell variability and visualize rare events, DNA combing-based RFD profiling is therefore a powerful new tool to investigate the replication of metazoan genomes.

## MATERIALS AND METHODS

### Biological resources

Chicken DT40 cells were purchased from the American Type Culture Collection (ATCC, #CRL-2111). DMD^Tet/Tet^ and CCSER1^βa/βa^ cells come from (46).

### Cell culture

DT40 cells were grown in RPMI 1640 medium (Gibco) with 10% fetal bovine serum (Gibco), 1% chicken serum (Sigma), 0.05 mM β-mercaptoethanol, 100 U ml^−1^ penicillin and 100 μg ml^−1^ streptomycin (Gibco) at 37°C, 20% O_2_, 5% CO_2_.

### RFD profiling by OK-seq

RFD profiling of DT40 cells by OK-seq was performed as described in (11) and (14). Briefly, asynchronously growing cells were labelled with 20 μM 5-ethynyl-2′-deoxyuridine (EdU, Jenabioscience) for 2 min. After DNA purification and heat-denaturation, Okazaki fragments were size-purified on sucrose gradients, labelled with biotin at EdU sites using click-chemistry, captured on magnetic beads coated with streptavidin and amplified by PCR. Sequencing was performed on HiSeq (Illumina) at the IB2C high-throughput sequencing platform (Gif-sur-Yvette, France). Aligned OK-seq data (BAM files) were imported in R (52) as *GenomicAlignments*, and reads were converted into *GenomicRanges*. RFD was computed using R as the difference between rightward- and leftward-fork coverage normalized by total coverage. Data were binned into nonoverlapping 10 kb windows. RFD profiles were exported into bigwig files using the export function from the *rtracklayer* package. The OK-seq experiment was performed three times. RFD profiles of the three biological replicates were highly similar, with Spearman's pairwise correlation coefficients computed in 10 kb windows ranging from 0.972 to 0.986. *DMD* and *CCSER1* OK-seq-based RFD profiles for the three replicates are presented in Supplementary Figure S2.

### Single molecule-based RFD and coverage profiles of *DMD* and *CCSER1* loci

**Figure 1.**
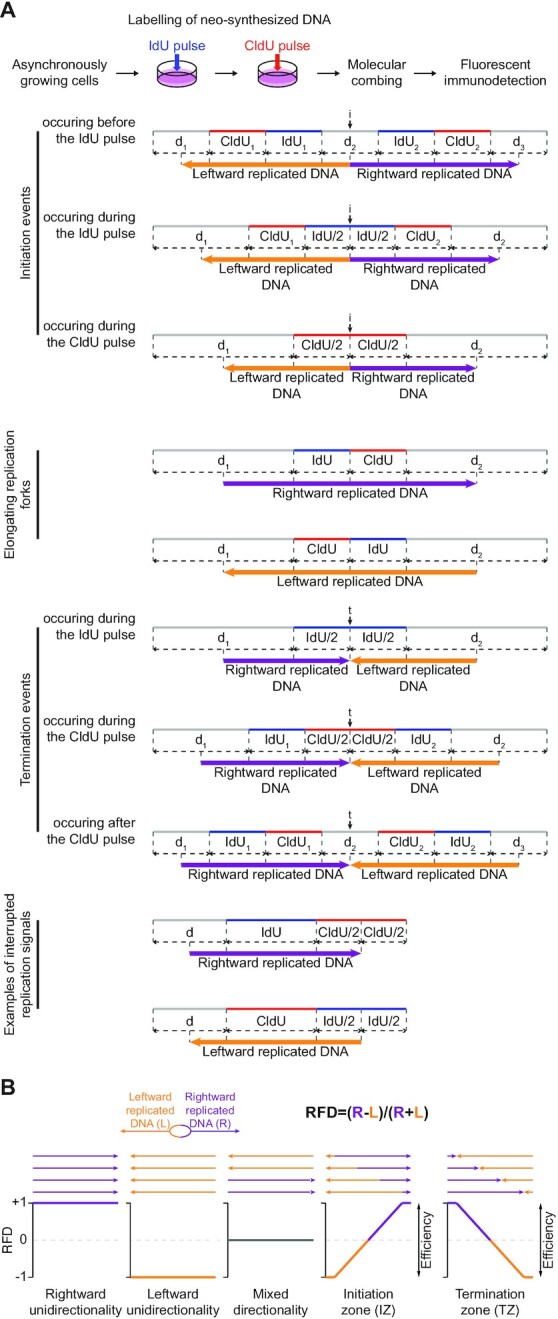
DNA combing-based RFD profiling. (**A**) Principles of replicated DNA directionality analyses. The protocol used in (46) for consecutive pulse-labelling of replication in asynchronously growing cells is presented on top. Below are schemes of the typical replication patterns visualized on combed molecules after immunodetection. Newly synthesized DNA labelled with IdU and CldU is represented by blue and red tracks, respectively; DNA fibres are in gray. Consecutive pulses with IdU and CldU discriminate between elongating replication forks, initiation (i) and termination (t) events, and enable determination for each DNA fibre mapped on a reference genome of the length of DNA replicated by rightward- and leftward-moving forks (purple and orange arrows, respectively), as illustrated. See text for details. IdU_i_ and CldU_*i*_ (*i* = ø, 1 or 2), length of the IdU and CldU tracks, respectively; *d_i_* (*i* = ø, 1, 2 or 3), length of the indicated unlabelled DNA fibre section (NB: *i* = object index when several similar objects, namely ‘IdU’, ‘CldU’ or ‘d’ are on the same DNA molecule). Black vertical arrows indicate the estimated positions of initiation and termination events. (**B**) Principles of RFD profiling. RFD is calculated for a given position as the difference between the proportions of rightward- and leftward-replicated DNA. Examples of theoretical situations with the corresponding RFD profiles are presented. The amplitude of the RFD shifts reflects the IZ and TZ efficiency.

The length of rightward- and leftward-replicated DNA was determined as described in Figure 1A for each DNA fibre with replication signals spanning *DMD, DMD^Tet^*, *CCSER1* or *CCSER1^β^^a^* obtained by combing and schematized in Supplementary Figure 4 of (46); examples of raw DNA combing data with replication and FISH signals on a DNA molecule are shown in Supplementary Figure 3 of (46). Coordinates, length and directionality of replicated DNA were compiled using Microsoft Excel. Coverages of rightward- (R) and leftward-replicated DNA (L) along *DMD* and *CCSER1* loci were determined and RFD was computed for each position as follows: RFD = (R − L)/(R + L) using custom R scripts. Initiation and termination zones were annotated by manually scanning the profiles.

### Individual initiation and termination events inside *DMD* and *CCSER1* loci

**Figure 2.**
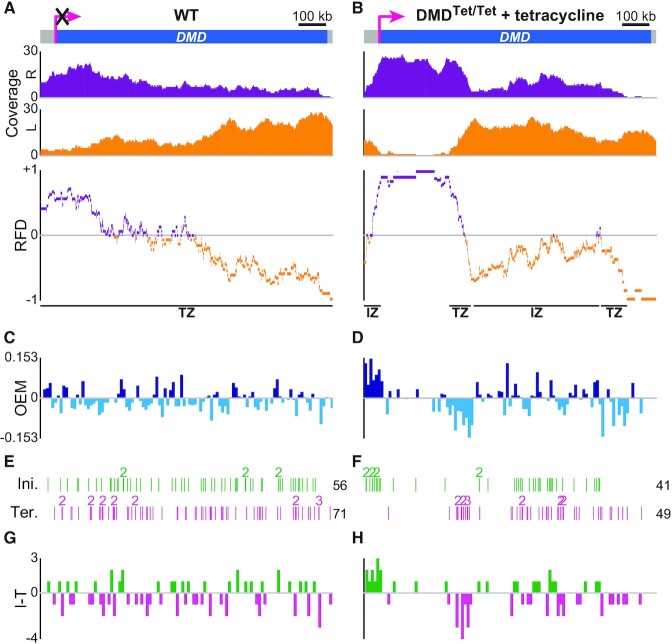
DNA combing-based RFD profiling of transcriptionally inactive and active *DMD* in DT40 cells. (A, B) RFD profile of *DMD* in WT cells (**A**) and DMD^Tet/Tet^ cells with tetracycline (**B**). From top to bottom: *DMD* locus (pink arrow, active promoter; the superimposed black cross indicates that WT *DMD* is not transcribed); coverages of rightward- (R, purple) and leftward-replicated DNA (L, orange); RFD profile, with manually annotated IZs and TZs delimited by black lines to guide the eyes. Data were computed from Supplementary Figure 4A in (46). 279 (mean size 85.9 kb) and 240 (mean size 103.8 kb) oriented tracks were aggregated to compute the RFD profile of *DMD* in WT cells and DMD^Tet/Tet^ cells with tetracycline, respectively. (C, D) OEM computed in 10 kb windows from the RFD profile of *DMD* in WT cells (**C**) and DMD^Tet/Tet^ cells with tetracycline (**D**). (E, F) Map of initiation (Ini.) and termination (Ter.) events along *DMD* in WT cells (**E**) and DMD^Tet/Tet^ cells with tetracycline (**F**) (data from Figure 1C, D in (46)). Each vertical line corresponds to the position of one initiation or termination event; numbers indicate colocalized events; the total number of events is indicated on the right. Because RFD profiles were strictly computed within the limits of the probes used to identify combed DNA molecules spanning *DMD*, the very limited number of events situated outside of those boundaries are not represented, which explains why the total number of initiation and termination events is sometimes slightly lower than in (46). (G, H) Density profile of initiation minus termination events (I − T) in 10 kb windows of *DMD* in WT cells (**G**) and DMD^Tet/Tet^ cells with tetracycline (**H**).

**Figure 3.**
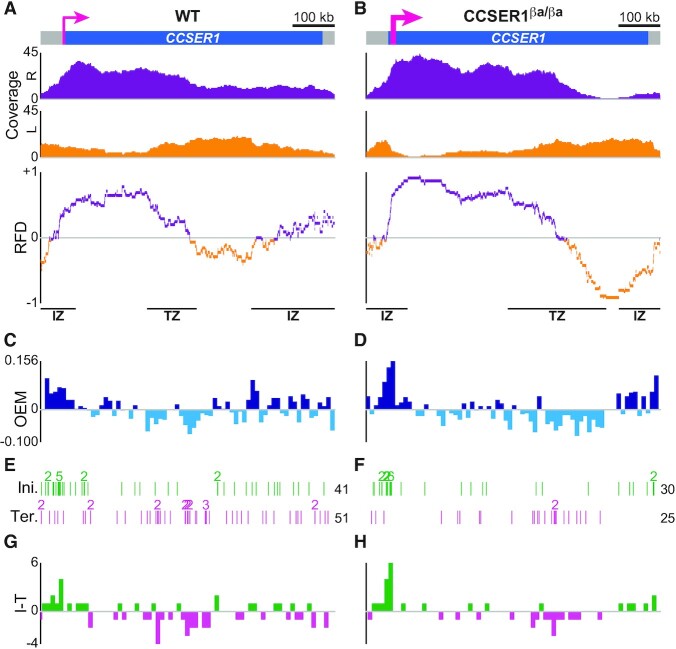
DNA combing-based RFD profiling of WT and overexpressed *CCSER1* in DT40 cells. (A, B) RFD profile of *CCSER1* in WT (**A**) and CCSER1^βa/βa^ cells (**B**). From top to bottom: *CCSER1* locus (thin and thick pink arrows, active *CCSER1* promoter in WT cells and active β-actin promoter in CCSER1^βa/βa^ cells, respectively); coverages of rightward- (R, purple) and leftward-replicated DNA (L, orange); RFD profile, with manually annotated IZs and TZs delimited by black lines to guide the eyes. Data were computed from Supplementary Figure 4B in (46). 211 (mean size 97.4 kb) and 240 (mean size 88.6 kb) oriented tracks were aggregated to compute the RFD profile of *CCSER1* in WT and CCSER1^βa/βa^ cells, respectively. (C, D) OEM computed in 10 kb windows from the RFD profile of *CCSER1* in WT (**C**) and CCSER1^βa/βa^ cells (**D**). (E, F) Map of initiation (Ini.) and termination (Ter.) events along *CCSER1* in WT (**E**) and CCSER1^βa/βa^ cells (**F**) (data from Figure 2C, D in (46)). See Figure 2E for details. (G, H) Density profile of initiation minus termination events (I − T) in 10 kb windows of *CCSER1* in WT (**G**) and CCSER1^βa/βa^ cells (**H**).

Positions of individual initiation and termination events in Figures 2E, F and 3E, F are from Figures 1C, D and 2C, D in (46), respectively.

### Fork speed distribution in *DMD* and *CCSER1* loci

The position and velocity of rightward and leftward forks progressing along *DMD* and *CCSER1* in WT and mutant cells were estimated from Supplementary Figure 4 of (46). Speed was determined for individual forks given a 20 minutes labelling time for both IdU and CldU as described in (46). Fork position corresponded either to: (i) the centre of the IdU and CldU tracks (*d*_IdU_ + *d*_CldU_)/2, with *d*_IdU_ and *d*_CldU_ being the lengths of the IdU and CldU tracks of the same fork, respectively, for forks displaying an intact IdU track flanked on one side by an intact CldU track; (ii) the centre of the IdU track for forks with an intact IdU signal flanked on one side by an interrupted CldU signal and (iii) the centre of the CldU track for forks with an intact CldU signal flanked on one side by an interrupted IdU signal. The interrupted signals reflect a physical break in the DNA since no underlying DNA was observed after immunodetection, in which case only the unbroken half of the signal track was used for the RFD computation, as illustrated in Figure 1A.

### (I − T) and OEM profiles

OEM (Origin Efficiency Metric) was computed as described in (48) from the DNA combing oriented tracks on 10 kb sliding (with 10 kb step) windows and the (I − T) density profile was calculated by binning initiation minus termination events in non-overlapping 10 kb windows using custom R scripts.

### Subsampling of OK-seq data

**Figure 4.**
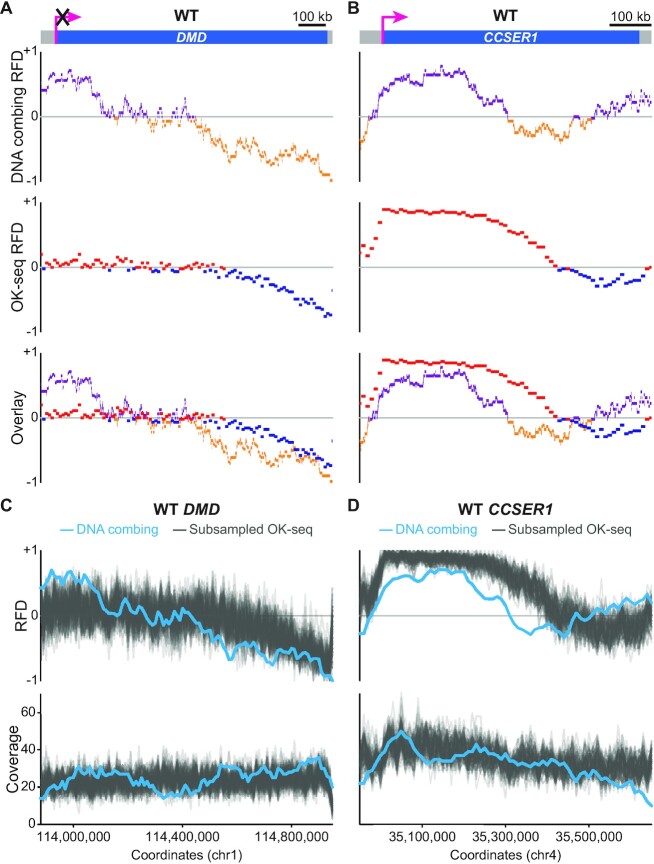
Comparison of DNA combing- and OK-seq-based RFD profiles of WT *DMD* and *CCSER1*. (A, B) Comparative analysis of the RFD profiles of WT *DMD* (**A**) and *CCSER1* (**B**) obtained by both methods. From top to bottom: locus represented as in Figures 2 or 3; RFD profile from DNA combing data; RFD profile from OK-seq data binned into 10 kb adjacent windows; overlay of the two profiles. The OK-seq experiment was performed three times with similar results; one representative profile is presented for the *DMD* and *CCSER1* loci. (C, D) Assessment of the sampling effect for WT *DMD* and *CCSER1* DNA combing-based RFD computation. One hundred RFD profiles were computed from OK-seq reads randomly subsampled to obtain a coverage comparable to that of WT *DMD* (**C**) or *CCSER1* (**D**) DNA combing analysis (see Material and Methods for details). From top to bottom: RFD profile from DNA combing data (light blue line) and 100 subsampled RFD profiles from OK-seq data (gray lines) binned into adjacent 10 kb windows; coverages of the DNA combing- and subsampled OK-seq-based RFD profiles (light blue and grey lines, respectively).

For subsampling, 2500 reads mapping on the locus of interest (either *DMD* or *CCSER1*) were selected randomly from the OK-seq dataset with the highest number of reads and used to compute RFD and coverage profiles using bins of 10 kb. This was performed 100 times and the 100 subsampled profiles are presented in Figure 4C, D along with the RFD and coverage from the corresponding single molecule data with the same binning interval.

### Visualisation tools

RFD profiles and graphical representations of coverages were made using Integrative Genomics Viewer (IGV) v2.8.10 or custom R scripts.

### Statistical analyses

Spearman's pairwise correlations between the RFD profiles of OK-seq biological replicates, between the DNA combing- and OK-seq-based RFD profiles binned into 10 kb windows and between the (I − T) and OEM profiles were computed with binned correlation function based on the R *cor* function.

### Genomic coordinates

Coordinates are given according to the ICGSC/galGal4 chicken genome assembly.

## RESULTS

### Principles of DNA combing-based RFD profiling

The principles of directionality measurement for all the replication signals visualized on combed DNA molecules following a consecutive pulse-labelling of cells with iododeoxyuridine (IdU) then chlorodeoxyuridine (CldU) are presented in Figure 1A. In DNA combing experiments, the positions of initiation and termination events are defined as the midpoints between diverging and converging forks, respectively. This is based on observations that, in most cases, diverging forks emanating from one origin, as well as converging forks coming from neighbouring origins, travel at a similar speed in mammalian cells (32,53). For an initiation event, the directionality of replicated DNA can therefore be determined starting from the midpoint between two diverging forks (i.e. the estimated position of the initiation event); conversely, for a termination event, directionality can be determined up to the midpoint between two converging forks (i.e. the estimated position of the termination event). It follows that, for an elongating replication fork moving to the right, rightward-replicated DNA extends from the midpoint between the start of the DNA fibre and the start of the IdU track up to the midpoint between the end of the DNA molecule and the end of the CldU track. The first midpoint corresponds to the estimated position of a hypothetical initiation event in case the DNA fibre was broken during the combing procedure precisely at the site of IdU incorporation for the diverging fork; replication directionality can no longer be unambiguously established beyond this point as the rightward fork might have emanated from a more distant origin. In the same line, replication directionality can only be assigned up to the second midpoint since the rightward fork may either continue its progression or merge with a converging fork beyond this position. The reasoning is similar for a leftward elongating fork: leftward-replicated DNA extends from the midpoint between the start of the DNA fibre and the start of the CldU track up to the midpoint between the end of the DNA molecule and the end of the IdU track.

RFD is computed as the difference between the proportions of rightward- and leftward-replicated DNA (Figure 1B). RFD ranges from −1 (100% of leftward-replicated DNA) to +1 (100% of rightward-replicated DNA). A null RFD means that DNA is replicated equally often by forks travelling leftward or rightward. As reported earlier, zones of predominant initiation (initiation zones, IZs) are detected as upward slopes on RFD profiles; conversely, downward slopes correspond to zones of predominant termination (termination zones, TZs) (11).

### DNA combing-based RFD profiles of transcriptionally inactive and active *DMD*

We first computed the RFD profile of *DMD* whether it is transcribed or not (Figure 2). A globally decreasing RFD was computed along the inactive *DMD* gene in WT cells (Figure 2A), with the downward slope reflecting a prevalence of termination over initiation in agreement with the total number of mapped individual termination (*n* = 71) and initiation (*n* = 56) events (Figure 2E). As previously indicated by the redistribution of initiation and termination events (46), activation of *DMD* transcription through the insertion of an inducible Tet-promoter dramatically reshuffled its replication dynamics, creating an extremely efficient IZ in 5′ of the gene followed by a large region where forks travelled almost exclusively rightward (Figure 2B). This region ended at a sharp TZ bordering a second, less efficient and broader IZ. The 3′ part of *DMD* was replicated predominantly leftward regardless of *DMD* transcriptional status (Figure 2A, B). Importantly, for active *DMD*, individual initiation and termination events clustered inside the IZs and TZs, respectively, as anticipated (Figure 2F). The unidirectional replication of the 5′ third of active *DMD* was also in good agreement with the paucity of initiation and termination events in that region. Moreover, given the duration of S-phase in DMD^Tet/Tet^ cells (46), the firing time of the IZ located at the 5′ end of active *DMD* (46) and the velocity of rightward forks emanating from that IZ (Supplementary Figure S1), we calculated that the first ≈300 kb of *DMD* should be unidirectionally replicated (Table 1), as observed on the RFD profile (Figure 2B).

**Table 1.** Calculation of the average distance travelled by rightward replication forks emanating from the IZ located in 5′ of *DMD^Tet^*. The 5′ IZ was considered to be fully efficient. Replication fork velocity was assumed to be constant throughout S-phase. fS1, fS2, fS3 and fS4 replication timing values correspond to the percentage of BrdU-labelled DNA relative to total S-phase in S1, S2, S3 and S4 fractions, respectively, at the position of the 5′ IZ (data from Figure 1F in (46); average percentage from two independent experiments). vRF, velocity of rightward forks

**Parameter**		**Value**	**Comments**
Doubling time		552 min	Data from Supplementary Figure 1G in ref. (46)
% of cells in S phase		69.4	Data from Supplementary Figure 1H in ref. (46)
Estimated S-phase duration		383 min	= % of cells in S phase*doubling time
Median velocity of rightward forks emanating from the IZ located in 5′ of *DMD^Tet^*		2.1 kb/min	vRF; see Supplementary Figure S1B
Theoretical distance travelled by rightward forks emanating from the IZ located in 5′ of *DMD^Tet^* starting in	S1	804 kb	dS1 = 1*S-phase duration*vRF
	S2	603 kb	dS2 = 0.75*S-phase duration*vRF
	S3	402 kb	dS3 = 0.5*S-phase duration*vRF
	S4	201 kb	dS4 = 0.25*S-phase duration*vRF
% of rightward forks emanating from the IZ located in 5′ of *DMD^Tet^* starting in	S1	2.2	fS1; data from Figure 1F in ref. (46)
	S2	6.2	fS2; data from Figure 1F in ref. (46)
	S3	26.2	fS3; data from Figure 1F in ref. (46)
	S4	65.4	fS4; data from Figure 1F in ref. (46)
**Average distance travelled by rightward forks emanating from the IZ located in 5′ of *DMD^Tet^***		**292 kb**	= dS1*fS1+dS2*fS2+dS3*fS3+dS4*fS4

RFD shifts over a defined region are mathematically predicted to be proportional to the difference between the number of initiation and termination events in this region (54). Accordingly, we found that the segments of the *DMD^Tet^* allele exhibiting steep RFD shifts, namely the very efficient 5′ IZ and the adjacent intragenic TZ, coincided with localized maxima and minima of initiation minus termination (I − T) values, respectively (Figure 2H). In parallel, we measured RFD slopes from the whole set of oriented tracks by computing the origin efficiency metric (OEM) profiles, corresponding to the difference of the normalized leftward coverage between adjacent 10 kb windows (48), of the WT and *DMD^Tet^* alleles (Figure 2C, D). The (I − T) and OEM profiles were comparable (Spearman's pairwise correlation coefficient ρ = 0.434 and 0.596 for *DMD* and *DMD^Tet^*, respectively; Figure 2C, G and D, H), highlighting the consistency between the analysis of individual events and the ensemble-averaging analysis of oriented replication tracks. The difference profile was less discriminating for segments of moderate RFD shifts, which presumably require a higher number of observations to accurately reflect population tendencies (compare for instance the OEM and (I − T) profiles of the 3′ TZ inside *DMD^Tet^* in Figure 2D, H). Despite the mapping of multiple initiation events inside the inactive *DMD* gene (Figure 2E), computation of initiation efficiency either from the oriented tracks or from the individual events did not identify regions of strong origin firing within the locus (Figure 2C, G), in line with the RFD pattern (Figure 2A).

Altogether, our results show that DNA combing-based RFD profiling goes beyond recapitulating the location of individual replication events. In addition to being a potent visual tool, it allows an in-depth analysis of the replication dynamics of a locus of interest, that is, the quantification of initiation, termination and fork progression; it also enables the cross-validation of orthogonally defined replication parameters. We found that initiation signals either occur in clusters associated with a positive RFD shift indicative of efficient usage in the cell population or as randomly distributed isolated events not accompanied by an upward slope on the RFD profile, confirming the existence of origins that are too rarely used to be detected by cell-population methods.

### DNA combing-based RFD profiling of *CCSER1* transcribed at different levels

We next examined *CCSER1* RFD profile in WT DT40 cells, where *CCSER1* is transcribed, and in CCSER1^βa/βa^ cells where *CCSER1* transcription was enhanced thanks to the insertion of the chicken β-actin promoter next to the endogenous one (46). The RFD profile of WT *CCSER1* showed a strong IZ upstream of the promoter and predominant rightward progression over the 5′ third of the gene, followed by a ≈150-kb-long TZ (Figure 3A). A broader, less efficient IZ was located at the 3′ end of *CCSER1*. Increasing *CCSER1* transcription enhanced the efficiency of both IZs, which caused an extension of the rightward replicated zone and the replication of the 3′ third of *CCSER1* by forks travelling almost exclusively leftward (Figure 3B). These results confirm our previous conclusion, based on the location of individual initiation events, that transcription promotes initiation both upstream and downstream of *CCSER1* (46).

There was a high concordance between IZ and TZ position and efficiency on *CCSER1* RFD profiles in WT and CCSER1^βa/βa^ cells and the location of previously mapped clusters of initiation and termination events, respectively (Figure 3E, F), as shown for active *DMD*. Consistently, for both the WT and *CCSER1^β^^a^* alleles, the (I − T) and OEM profiles were very much alike (Spearman's pairwise correlation coefficient ρ = 0.549 and 0.587, respectively; Figure 3C, G and D, H). However, some features revealed by RFD profiling were hardly predictable from the sole positioning of individual events, such as the broad, poorly efficient IZ at its 3′ end (Figure 3A, E). This further exemplifies that DNA combing-based RFD profiling can surpass classical analyses focusing on initiation and termination events to comprehend the replication program, particularly in regions of mixed directionality. The RFD profile of the *CCSER1^β^^a^* allele also demonstrated that it was primarily replicated by converging forks emanating from the IZs located at both ends of the gene and meeting two-thirds of the way into the *CCSER1* gene (Figure 3B), which was less clearly shown by the sole location of initiation and termination events (Figure 3F).

Interestingly, *CCSER1^β^^a^* RFD profile closely resembled the crenelated N-shaped RFD profile observed by Petryk and colleagues in the human genome (11) consisting of two ascending segments (the IZs in 5′ and 3′' of the *CCSER1^β^^a^* allele) followed by flat sections of high absolute value of RFD (the regions of *CCSER1^β^^a^* with predominant unidirectional fork progression) meeting within a descending segment (the TZ located at two thirds of *CCSER1^β^^a^*) (Figure 3B). Crenelated N-shaped RFD profiles are presumed to be created when two IZs are far and efficient enough to prevent the reaching of one IZ by forks emanating from the other one (11). Accordingly, taking into account the distance between the IZs in 5′ and 3′ of *CCSER1^β^^a^* (i.e. the length of the allele), their firing time (46) and the velocity of forks emanating from each IZ (Supplementary Figure S1), we computed that less than 10% of rightward forks emanating from the 5′ IZ could reach the 3′ IZ, whereas reaching of the 5′ IZ by forks coming from the 3′ IZ was virtually impossible (Table 2). Moreover, we calculated that rightward and leftward forks should merge on average ≈380 kb from the start of the *CCSER1^β^^a^* allele (Table 2), in good agreement with what was observed on the RFD profile (Figure 3B). In conclusion, our DNA combing-based RFD profiling method substantiated the replication mode of crenelated N-shaped RFD domains, herein exemplified by the *CCSER1^β^^a^* allele.

**Table 2.** Calculation of the average meeting point of rightward and leftward replication forks progressing along *CCSER1^β^^a^* and of the proportion of rightward (leftward) forks reaching the IZ located in 3′ (5′) of *CCSER1^β^^a^*. To reach the 3′ (5′) IZ, rightward (leftward) forks coming from the 5′ (3′) IZ must have travelled a minimum of 621 kb (*i.e*., *CCSER1^β^^a^* length) before the 3′ (5′) IZ fired, that is, at the latest, before S4. Only rightward (leftward) forks emanating from the 5′ (3′) IZ at the beginning of S-phase (S1 fraction), which could theoretically travel 663 (628) kb by the end of the third quarter of S-phase, fulfil these criteria. These forks accounted for 26.3 (5.7) % of total rightward (leftward) forks, while the 3′ (5′) IZ fired after S3 33.2 (2.9) % of the time. The 5′ and 3′ IZs were considered to be fully efficient. Replication fork velocity was assumed to be constant throughout S-phase. fS1 (fS1′), fS2 (fS2′), fS3 (fS3′) and fS4 (fS4′) replication timing values correspond to the percentage of BrdU-labelled DNA relative to total S-phase in S1, S2, S3 and S4 fractions, respectively, at the position of the 5′ (3′) IZ (data from Figure 2F in (46); average percentage from two independent experiments). vRF, velocity of rightward forks; vLF, velocity of leftward forks

**Parameter**		**Value**	**Comments**
Doubling time		744 min	Data from Supplementary Figure 5D in (46)
% of cells in S phase		62.5	Data from Supplementary Figure 5E in (46)
Estimated S-phase duration		465 min	= % of cells in S phase*doubling time
Median velocity of rightward forks emanating from the IZ located in 5′ of *CCSER1^β^^a^*		1.9 kb/min	vRF; see Supplementary Figure S1D
Theoretical distance travelled by rightward forks emanating from the IZ located in 5′ of *CCSER1^β^^a^* starting in	S1	884 kb	dS1 = 1*S-phase duration*vRF
	S2	663 kb	dS2 = 0.75*S-phase duration*vRF
	S3	442 kb	dS3 = 0.5*S-phase duration*vRF
	S4	221 kb	dS4 = 0.25*S-phase duration*vRF
% of rightward forks emanating from the IZ located in 5′ of *CCSER1^β^^a^* starting in	S1	26.3	fS1; data from Figure 2F in (46)
	S2	53.2	fS2; data from Figure 2F in (46)
	S3	17.6	fS3; data from Figure 2F in (46)
	S4	2.9	fS4; data from Figure 2F in (46)
Average distance potentially travelled by rightward forks emanating from the IZ located in 5′ of *CCSER1^β^^a^*		669 kb	dIZ1 = dS1*fS1+dS2*fS2+dS3*fS3+dS4*fS4
Median velocity of leftward forks emanating from the IZ located in 3′ of *CCSER1^β^^a^*		1.8 kb/min	vLF. See Supplementary Figure S1D
Theoretical distance travelled by lefttward forks emanating from the IZ located in 3′ of *CCSER1^β^^a^* starting in	S1	837 kb	dS1′ = 1*S-phase duration*vLF
	S2	628 kb	dS2′ = 0.75*S-phase duration*vLF
	S3	419 kb	dS3′ = 0.5*S-phase duration*vLF
	S4	209 kb	dS4′ = 0.25*S-phase duration*vLF
% of leftward forks emanating from the IZ located in 3′ of *CCSER1^β^^a^* starting in	S1	5.7	fS1′; data from Figure 2F in (46)
	S2	20.1	fS2′; data from Figure 2F in (46)
	S3	41.0	fS3′; data from Figure 2F in (46)
	S4	33.2	fS4′; data from Figure 2F in (46)
Average distance potentially travelled by leftward forks emanating from the IZ located in 3′ of *CCSER1^β^^a^*		415 kb	dIZ2 = dS1′*fS1′+dS2′*fS2′+dS3′*fS3′+dS4′*fS4′
*CCSER1^β^^a^* length		621 kb	Data from Supplementary Figure 5A in (46)
**Average meeting point of rightward and leftward progressing forks (distance from *CCSER1^β^^a^* TSS)**		**383 kb**	= dIZ1/(dIZ1+dIZ2)*length(*CCSER1^β^^a^)*
**% of rightward forks emanating from the IZ located in 5′ of *CCSER1^β^^a^* able to reach the IZ located in 3′ of *CCSER1^β^^a^***		**8.7**	=fS1*fS4′*100
**% of leftward forks emanating from the IZ located in 3′ of *CCSER1^β^^a^* able to reach the IZ located in 5′ of *CCSER1^β^^a^***		**0.17**	=fS4*fS1′*100

### Comparison of DNA combing-based RFD profiles with population-based RFD analyses

To further validate our SM profiling method, we compared the DNA combing-based RFD profiles of WT *DMD* and *CCSER1* to independent RFD profiles obtained by the sequencing of ethynyldeoxyuridine-labelled Okazaki fragments purified from DT40 cells (Figure 4A, B and Supplementary Figure S2). *DMD* profiles from DNA combing and from each of the three biological replicates generated for OK-seq were highly similar (Spearman's pairwise correlation coefficient ranging from 0.812 to 0.831), demonstrating the accuracy of our approach. Of note, the RFD profile of the 5′-most part of *DMD* computed from OK-seq data was flatter than the profile assembled from single molecules (Figure 4A), which was likely explained by a sampling effect for the latter (Figure 4C). The number of oriented tracks (*n* = 279) aggregated for WT *DMD* DNA combing-based RFD computation indeed remained drastically low compared to the throng of Okazaki fragments (*n* ≈ 60 000) used to build the OK-seq profile, which certainly provided a better estimate of the average RFD. We also observed that WT *CCSER1* DNA combing- and OK-seq-based RFD profiles were largely comparable (Spearman's pairwise correlation coefficient ranging from 0.645 to 0.657 depending on the OK-seq sample; Figure 4B), although not completely superimposable. In contrast to *DMD*, the observed discrepancies seemed not to be solely due to a sampling effect for the DNA combing-based RFD profile (Figure 4D), and their origin remains unclear. The visibly increased efficiency of the IZ upstream of *CCSER1* in the OK-seq conditions would fully explain the extension of the region of predominant rightward fork progression, the shift in the TZ location and the partial suppression of the 3′ IZ compared to the DNA combing profile.

## DISCUSSION

A seminal study by Czajkowsky and colleagues first demonstrated that the stacking of a large number of individual replication signals reproduces population-averaging profiles (55). Very recently, the FORK-seq method yielded an RFD profile of the yeast genome assembled from thousands of oriented replication tracks that was indistinguishable from that produced by the sequencing of Okazaki fragments (36). In higher eukaryotes, only a few SM-based replication analyses, for instance those using the SMARD (single molecule analysis of replicated DNA) technique (56), went beyond the usual mapping of individual replication signals. And yet, whereas the successive incorporation of IdU then CldU only gives a relative direction of fork progression on anonymous DNA fibres the orientation of which remains elusive, FISH-based positioning of stretched DNA molecules on a reference genome allows correct orientation of each replication track and permits reliable quantification of RFD provided that a sufficient number of oriented tracks are aggregated. As a proof of principle, using the DNA fibres collected in our previous study (46), we computed the RFD profiles of two large genes, namely *DMD* and *CCSER1*, each in different transcriptional states, from the hundreds of oriented replication tracks spanning each locus. Importantly, these profiles are consistent with the location of individual initiation and termination events, with measured replication fork velocities as well as with replication timing analyses. They are also concordant with RFD profiles produced by OK-seq, reciprocally validating each other. Altogether, these results demonstrate the accuracy of our method, although discrepancies between DNA combing and OK-seq RFD profiles are observed for both WT *DMD* and *CCSER1*. In the case of *DMD*, differences seem largely attributable to a sampling effect for the DNA combing-based profile due to the relatively low number of aggregated oriented tracks. However, this cannot explain the disparities for *CCSER1*. Intriguingly, *CCSER1* OK-seq profile resembles the DNA combing profile of the *CCSER1^β^^a^* allele (i.e. of overexpressed *CCSER1*), suggesting that *CCSER1* transcription level might have been higher when DT40 cells were grown for OK-seq than when they were cultured for DNA combing analyses. In this regard, although cells used for either method were grown in distinct laboratories and over a time window that precluded the use of the exact same culture medium, the obtained RFD profiles are still globally similar (average Spearman's pairwise correlation coefficient of 0.822 and 0.651 for *DMD* and *CCSER1*, respectively), confirming the robustness of our approach.

By granting access to fork velocity, cell-to-cell heterogeneity and replication events invisible to population assays, DNA combing-based RFD profiles allow the interrogation of models built on ensemble-averaging techniques. We could substantiate the mode of replication of crenelated N-shaped RFD domains initially detected on RFD profiles of the human genome (11). Moreover, the detection of randomly distributed initiation events in WT *DMD* near-horizontal and descending RFD sections unambiguously establishes that initiation is not solely limited to the ascending segments bordering RFD domains, that is, to IZs. We therefore propose that the replication of metazoan genomes associates strong initiation in delimited zones with widely dispersed, low efficiency origins elsewhere, as previously suggested (11). The mapping of initiation events unable to appear as detectable RFD upward shifts likely explains long-standing discrepancies between cell-population and SM methods regarding the number of active origins per genome, for example why only 5684 IZs were counted in human lymphoblasts (11) whereas over three times more could have been expected according to the ≈150 kb inter-origin distance measured by DNA fibre analysis in those cells (32). In line with this, a recent high-resolution replication timing profiling technique found that >70% of the genome of mammalian cells host initiation sites although <10% are constituted by IZs (57). Finally, our approach makes it possible to explain if an observed variation in RFD is due either to a change in initiation, a change in termination, or both, and to decide between several scenarios that could all explain a given RFD pattern. Notably, DNA combing-based RFD profiling demonstrates that descending segments correspond either to regions of nearly exclusive fork fusion, as inside *CCSER1^β^^a^*, in agreement with the ‘two-origin’ model suggesting that replication initiation is restricted to the borders of RFD domains (58), or to regions displaying a combination of initiation and termination events, as in the case of WT *DMD*, reminiscent of the ‘cascade’ model of sequential origin activation with increasing synchrony from RFD domain borders to center (11,45,59). It is anticipated that initiation within RFD domains only becomes mandatory when these domains are too large to be replicated by forks emanating exclusively from the bordering IZs, which depends on fork lifespan and velocity. Consistently, we computed that the ≈600-kb-long crenelated N-shaped RFD domain made of *CCSER1^β^^a^* can be entirely replicated by converging forks (Table 2), and therefore does not require additional initiation events.

In conclusion, besides providing a good proxy for population-averaged RFD from a limited number of replication tracks, our DNA combing-based replication profiling method has the unique ability to identify discrete events on individual DNA molecules, helping to reconcile SM- and population-based assays. There is little doubt that similar analyses will shortly be performed on complete metazoan genomes using either nanopore sequencing-based techniques or optical replication mapping. While this manuscript was in preparation, a preprint (41) actually reported the optical mapping of millions of single DNA molecules from synchronized human cells electroporated with a fluorescent dUTP at the beginning of S-phase, thereby identifying early-firing initiation sites genome-wide. Replication tracks from fluorescent dUTP-labelled asynchronously growing cells were also mapped, and track polarity was inferred thanks to the decreasing label density as electroporated nucleotides were consumed, allowing to approximate an RFD profile of the human genome. Although still preliminary, this work illustrates the recent impressive progress in DNA fibre analysis, with the promise of elucidating the DNA replication strategy of higher eukaryotes.

## DATA AVAILABILITY

OK-Seq data for *DMD* and *CCSER1* loci in DT40 cells have been deposited at NCBI’s Gene Expression Omnibus (GEO) repository and are accessible under accession number GSE164252. The datasets generated during the current study, including bed files with DT40 OK-seq reads for WT *CCSER1* and *DMD* loci, oriented tracks, initiation and termination events from DNA combing analyses, as well as custom R scripts are available from the github repository (https://github.com/LacroixLaurent/DT40_RFD).

## Supplementary Material

gkab219_Supplemental_File

## Notes

Present address: Nataliya Petryk, Epigenetics and Cell Fate, UMR7216 CNRS, University of Paris, F-75013 Paris, France.

## References

[1] Hyrien O. Peaks cloaked in the mist: the landscape of mammalian replication origins. J. Cell Biol. 2015; 208:147–160. 25601401 10.1083/jcb.201407004 PMC4298691

[2] Urban J.M. , Foulk M.S., Casella C., Gerbi S.A. The hunt for origins of DNA replication in multicellular eukaryotes. F1000Prime Rep. 2015; 7:30. 25926981 10.12703/P7-30 PMC4371235

[3] Prioleau M.N. , MacAlpine D.M. DNA replication origins-where do we begin?. Genes Dev. 2016; 30:1683–1697. 27542827 10.1101/gad.285114.116 PMC5002974

[4] Cadoret J.C. , Meisch F., Hassan-Zadeh V., Luyten I., Guillet C., Duret L., Quesneville H., Prioleau M.N. Genome-wide studies highlight indirect links between human replication origins and gene regulation. Proc. Natl. Acad. Sci. U.S.A. 2008; 105:15837–15842. 18838675 10.1073/pnas.0805208105 PMC2572913

[5] Sequeira-Mendes J. , Diaz-Uriarte R., Apedaile A., Huntley D., Brockdorff N., Gomez M. Transcription initiation activity sets replication origin efficiency in mammalian cells. PLos Genet. 2009; 5:e1000446. 19360092 10.1371/journal.pgen.1000446 PMC2661365

[6] Karnani N. , Taylor C.M., Malhotra A., Dutta A. Genomic study of replication initiation in human chromosomes reveals the influence of transcription regulation and chromatin structure on origin selection. Mol. Biol. Cell. 2010; 21:393–404. 19955211 10.1091/mbc.E09-08-0707 PMC2814785

[7] Martin M.M. , Ryan M., Kim R., Zakas A.L., Fu H., Lin C.M., Reinhold W.C., Davis S.R., Bilke S., Liu H. et al. Genome-wide depletion of replication initiation events in highly transcribed regions. Genome Res. 2011; 21:1822–1832. 21813623 10.1101/gr.124644.111 PMC3205567

[8] Cayrou C. , Coulombe P., Vigneron A., Stanojcic S., Ganier O., Peiffer I., Rivals E., Puy A., Laurent-Chabalier S., Desprat R. et al. Genome-scale analysis of metazoan replication origins reveals their organization in specific but flexible sites defined by conserved features. Genome Res. 2011; 21:1438–1449. 21750104 10.1101/gr.121830.111 PMC3166829

[9] Besnard E. , Babled A., Lapasset L., Milhavet O., Parrinello H., Dantec C., Marin J.M., Lemaitre J.M. Unraveling cell type-specific and reprogrammable human replication origin signatures associated with G-quadruplex consensus motifs. Nat. Struct. Mol. Biol. 2012; 19:837–844. 22751019 10.1038/nsmb.2339

[10] Pratto F. , Brick K., Cheng G., Lam G., Cloutier J.M., Dahiya D., Wellard S.R., Jordan P.W., Camerini-Otero R.D. Germline DNA replication shapes the recombination landscape in mammals. 2020; bioRxiv doi:23 September 2020, preprint: not peer reviewed 10.1101/2020.09.23.308874. PMC8591710 34260899

[11] Petryk N. , Kahli M., Aubenton-Carafa Y., Jaszczyszyn Y., Shen Y., Silvain M., Thermes C., Chen C.L., Hyrien O. Replication landscape of the human genome. Nat. Commun. 2016; 7:10208. 26751768 10.1038/ncomms10208 PMC4729899

[12] Pourkarimi E. , Bellush J.M., Whitehouse I. Spatiotemporal coupling and decoupling of gene transcription with DNA replication origins during embryogenesis in C. elegans. 2016; 5:e21728. 10.7554/eLife.21728 PMC5222557 28009254

[13] Tubbs A. , Sridharan S., van Wietmarschen N., Maman Y., Callen E., Stanlie A., Wu W., Wu X., Day A., Wong N. et al. Dual roles of poly(dA:dT) tracts in replication initiation and fork collapse. Cell. 2018; 174:1127–1142. 30078706 10.1016/j.cell.2018.07.011 PMC6591735

[14] Wu X. , Kabalane H., Kahli M., Petryk N., Laperrousaz B., Jaszczyszyn Y., Drillon G., Nicolini F.E., Perot G., Robert A. et al. Developmental and cancer-associated plasticity of DNA replication preferentially targets GC-poor, lowly expressed and late-replicating regions. Nucleic Acids Res. 2018; 46:10532. 30212853 10.1093/nar/gky849 PMC6212835

[15] Petryk N. , Dalby M., Wenger A., Stromme C.B., Strandsby A., Andersson R., Groth A. MCM2 promotes symmetric inheritance of modified histones during DNA replication. Science. 2018; 361:1389–1392. 30115746 10.1126/science.aau0294

[16] Chen Y.H. , Keegan S., Kahli M., Tonzi P., Fenyo D., Huang T.T., Smith D.J. Transcription shapes DNA replication initiation and termination in human cells. Nat. Struct. Mol. Biol. 2019; 26:67–77. 30598550 10.1038/s41594-018-0171-0 PMC6320713

[17] Sriramachandran A.M. , Petrosino G., Mendez-Lago M., Schafer A.J., Batista-Nascimento L.S., Zilio N., Ulrich H.D. Genome-wide nucleotide-resolution mapping of DNA replication patterns, single-strand breaks, and lesions by GLOE-Seq. Mol. Cell. 2020; 78:975–985. 32320643 10.1016/j.molcel.2020.03.027 PMC7276987

[18] Kara N. , Krueger F., Rugg-Gunn P., Houseley J. Genome-wide analysis of DNA replication and DNA double strand breaks by TrAEL-seq. 2020; bioRxiv doi:10 August 2020, preprint: not peer reviewed 10.1101/2020.08.10.243931. PMC8021198 33760805

[19] Li Z. , Hua X., Serra-Cardona A., Xu X., Gan S., Zhou H., Yang W.S., Chen C.L., Xu R.M., Zhang Z. DNA polymerase alpha interacts with H3-H4 and facilitates the transfer of parental histones to lagging strands. Sci. Adv. 2020; 6:eabb5820. 32923642 10.1126/sciadv.abb5820 PMC7449674

[20] Mesner L.D. , Valsakumar V., Karnani N., Dutta A., Hamlin J.L., Bekiranov S. Bubble-chip analysis of human origin distributions demonstrates on a genomic scale significant clustering into zones and significant association with transcription. Genome Res. 2011; 21:377–389. 21173031 10.1101/gr.111328.110 PMC3044852

[21] Mesner L.D. , Valsakumar V., Cieslik M., Pickin R., Hamlin J.L., Bekiranov S. Bubble-seq analysis of the human genome reveals distinct chromatin-mediated mechanisms for regulating early- and late-firing origins. Genome Res. 2013; 23:1774–1788. 23861383 10.1101/gr.155218.113 PMC3814878

[22] MacAlpine H.K. , Gordan R., Powell S.K., Hartemink A.J., MacAlpine D.M. Drosophila ORC localizes to open chromatin and marks sites of cohesin complex loading. Genome Res. 2010; 20:201–211. 19996087 10.1101/gr.097873.109 PMC2813476

[23] Dellino G.I. , Cittaro D., Piccioni R., Luzi L., Banfi S., Segalla S., Cesaroni M., Mendoza-Maldonado R., Giacca M., Pelicci P.G. Genome-wide mapping of human DNA-replication origins: levels of transcription at ORC1 sites regulate origin selection and replication timing. Genome Res. 2013; 23:1–11. 23187890 10.1101/gr.142331.112 PMC3530669

[24] Miotto B. , Ji Z., Struhl K. Selectivity of ORC binding sites and the relation to replication timing, fragile sites, and deletions in cancers. Proc. Natl. Acad. Sci. U.S.A. 2016; 113:E4810–E4819. 27436900 10.1073/pnas.1609060113 PMC4995967

[25] Sugimoto N. , Maehara K., Yoshida K., Ohkawa Y., Fujita M. Genome-wide analysis of the spatiotemporal regulation of firing and dormant replication origins in human cells. Nucleic Acids Res. 2018; 46:6683–6696. 29893900 10.1093/nar/gky476 PMC6061783

[26] Kirstein N. , Buschle A., Wu X., Krebs S., Blum H., Hammerschmidt W., Lacroix L., Hyrien O., Audit B., Schepers A. Human ORC/MCM density is low in active genes and correlates with replication time but does not delimit initiation zones. Elife. 2021; 10:e62161. 33683199 10.7554/eLife.62161 PMC7993996

[27] Langley A.R. , Graf S., Smith J.C., Krude T. Genome-wide identification and characterisation of human DNA replication origins by initiation site sequencing (ini-seq). Nucleic Acids Res. 2016; 44:10230–10247. 27587586 10.1093/nar/gkw760 PMC5137433

[28] Macheret M. , Halazonetis T.D. Intragenic origins due to short G1 phases underlie oncogene-induced DNA replication stress. Nature. 2018; 555:112–116. 29466339 10.1038/nature25507 PMC5837010

[29] Foss E.J. , Sripathy S., Gatbonton-Schwager T., Kwak H., Thiesen A.H., Lao U., Bedalov A. Chromosomal Mcm2-7 distribution is the primary driver of the genome replication program in species from yeast to humans. 2020; bioRxiv doi:26 April 2020, preprint: not peer reviewed 10.1101/737742. PMC8443269 34473702

[30] Kumagai A. , Dunphy W.G. Binding of the Treslin-MTBP complex to specific regions of the human genome promotes the initiation of DNA replication. Cell Rep. 2020; 32:108178. 32966791 10.1016/j.celrep.2020.108178 PMC7523632

[31] Bianco J.N. , Poli J., Saksouk J., Bacal J., Silva M.J., Yoshida K., Lin Y.-L., Tourrière H., Lengronne A., Pasero P. Analysis of DNA replication profiles in budding yeast and mammalian cells using DNA combing. Methods. 2012; 57:149–157. 22579803 10.1016/j.ymeth.2012.04.007

[32] Techer H. , Koundrioukoff S., Azar D., Wilhelm T., Carignon S., Brison O., Debatisse M., Le Tallec B. Replication dynamics: biases and robustness of DNA fiber analysis. J. Mol. Biol. 2013; 425:4845–4855. 23557832 10.1016/j.jmb.2013.03.040

[33] Bialic M. , Coulon V., Drac M., Gostan T., Schwob E. Vengrova S. , Dalgaard J. Analyzing the dynamics of DNA replication in mammalian cells using DNA combing. DNA Replication. Methods in Molecular Biology. 2015; 1300:NY Humana Press 67–78. 10.1007/978-1-4939-2596-4_4 25916705

[34] Lebofsky R. , Heilig R., Sonnleitner M., Weissenbach J., Bensimon A. DNA replication origin interference increases the spacing between initiation events in human cells. Mol. Biol. Cell. 2006; 17:5337–5345. 17005913 10.1091/mbc.E06-04-0298 PMC1679695

[35] Muller C.A. , Boemo M.A., Spingardi P., Kessler B.M., Kriaucionis S., Simpson J.T., Nieduszynski C.A. Capturing the dynamics of genome replication on individual ultra-long nanopore sequence reads. Nat. Methods. 2019; 16:429–436. 31011185 10.1038/s41592-019-0394-y PMC7617212

[36] Hennion M. , Arbona J.M., Lacroix L., Cruaud C., Theulot B., Tallec B.L., Proux F., Wu X., Novikova E., Engelen S. et al. FORK-seq: replication landscape of the Saccharomyces cerevisiae genome by nanopore sequencing. Genome Biol. 2020; 21:125. 32456659 10.1186/s13059-020-02013-3 PMC7251829

[37] Georgieva D. , Liu Q., Wang K., Egli D. Detection of base analogs incorporated during DNA replication by nanopore sequencing. Nucleic. Acids. Res. 2020; 48:e88. 32710620 10.1093/nar/gkaa517 PMC7470954

[38] De Carli F. , Gaggioli V., Millot G.A., Hyrien O. Single-molecule, antibody-free fluorescent visualisation of replication tracts along barcoded DNA molecules. Int. J. Dev. Biol. 2016; 60:297–304. 27251072 10.1387/ijdb.160139oh

[39] Lacroix J. , Pelofy S., Blatche C., Pillaire M.J., Huet S., Chapuis C., Hoffmann J.S., Bancaud A. Analysis of DNA replication by optical mapping in nanochannels. Small. 2016; 12:5963–5970. 27624455 10.1002/smll.201503795

[40] De Carli F. , Menezes N., Berrabah W., Barbe V., Genovesio A., Hyrien O. High-throughput optical mapping of replicating DNA. Small Methods. 2018; 2:1800146.

[41] Wang W. , Klein K., Proesmans K., Yang H., Marchal C., Zhu X., Borrman T., Hastie A., Weng Z., Bechhoefer J. et al. Genome-wide mapping of human DNA replication by Optical Replication Mapping supports a stochastic model of eukaryotic replication. 2020; bioRxiv doi:18 September 2020, preprint: not peer reviewed 10.1101/2020.08.24.263459. PMC8286344 34157308

[42] Palumbo E. , Matricardi L., Tosoni E., Bensimon A., Russo A. Replication dynamics at common fragile site FRA6E. Chromosoma. 2010; 119:575–587. 20585795 10.1007/s00412-010-0279-4

[43] Letessier A. , Millot G.A., Koundrioukoff S., Lachages A.M., Vogt N., Hansen R.S., Malfoy B., Brison O., Debatisse M. Cell-type-specific replication initiation programs set fragility of the FRA3B fragile site. Nature. 2011; 470:120–123. 21258320 10.1038/nature09745

[44] Ozeri-Galai E. , Lebofsky R., Rahat A., Bester A.C., Bensimon A., Kerem B. Failure of origin activation in response to fork stalling leads to chromosomal instability at fragile sites. Mol. Cell. 2011; 43:122–131. 21726815 10.1016/j.molcel.2011.05.019

[45] Guilbaud G. , Rappailles A., Baker A., Chen C.L., Arneodo A., Goldar A., Aubenton-Carafa Y., Thermes C., Audit B., Hyrien O. Evidence for sequential and increasing activation of replication origins along replication timing gradients in the human genome. PLoS Comput. Biol. 2011; 7:e1002322. 22219720 10.1371/journal.pcbi.1002322 PMC3248390

[46] Blin M. , Le Tallec B., Nahse V., Schmidt M., Brossas C., Millot G.A., Prioleau M.N., Debatisse M. Transcription-dependent regulation of replication dynamics modulates genome stability. Nat. Struct. Mol. Biol. 2019; 26:58–66. 30598553 10.1038/s41594-018-0170-1

[47] Smith D.J. , Whitehouse I. Intrinsic coupling of lagging-strand synthesis to chromatin assembly. Nature. 2012; 483:434–438. 22419157 10.1038/nature10895 PMC3490407

[48] McGuffee S.R. , Smith D.J., Whitehouse I. Quantitative, genome-wide analysis of eukaryotic replication initiation and termination. Mol. Cell. 2013; 50:123–135. 23562327 10.1016/j.molcel.2013.03.004 PMC3628276

[49] Reijns M.A.M. , Kemp H., Ding J., de Proce S.M., Jackson A.P., Taylor M.S. Lagging-strand replication shapes the mutational landscape of the genome. Nature. 2015; 518:502–506. 25624100 10.1038/nature14183 PMC4374164

[50] Daigaku Y. , Keszthelyi A., Muller C.A., Miyabe I., Brooks T., Retkute R., Hubank M., Nieduszynski C.A., Carr A.M. A global profile of replicative polymerase usage. Nat. Struct. Mol. Biol. 2015; 22:192–198. 25664722 10.1038/nsmb.2962 PMC4789492

[51] Clausen A.R. , Lujan S.A., Burkholder A.B., Orebaugh C.D., Williams J.S., Clausen M.F., Malc E.P., Mieczkowski P.A., Fargo D.C., Smith D.J. et al. Tracking replication enzymology in vivo by genome-wide mapping of ribonucleotide incorporation. Nat. Struct. Mol. Biol. 2015; 22:185–191. 25622295 10.1038/nsmb.2957 PMC4351163

[52] The R Core Team R: a language and environment for statistical computing. R Foundation for Statistical Computing. 2020;

[53] Conti C. , Sacca B., Herrick J., Lalou C., Pommier Y., Bensimon A. Replication fork velocities at adjacent replication origins are coordinately modified during DNA replication in human cells. Mol. Biol. Cell. 2007; 18:3059–3067. 17522385 10.1091/mbc.E06-08-0689 PMC1949372

[54] Audit B. , Baker A., Chen C.-L., Rappailles A., Guilbaud G., Julienne H., Goldar A., Aubenton-Carafa Y., Hyrien O., Thermes C. et al. Multiscale analysis of genome-wide replication timing profiles using a wavelet-based signal-processing algorithm. Nat. Protoc. 2012; 8:98–110. 23237832 10.1038/nprot.2012.145

[55] Czajkowsky D.M. , Liu J., Hamlin J.L., Shao Z. DNA combing reveals intrinsic temporal disorder in the replication of yeast chromosome VI. J. Mol. Biol. 2008; 375:12–19. 17999930 10.1016/j.jmb.2007.10.046 PMC2151843

[56] Demczuk A. , Gauthier M.G., Veras I., Kosiyatrakul S., Schildkraut C.L., Busslinger M., Bechhoefer J., Norio P. Regulation of DNA replication within the immunoglobulin heavy-chain locus during B cell commitment. PLoS Biol. 2012; 10:e1001360. 22807655 10.1371/journal.pbio.1001360 PMC3393677

[57] Zhao P.A. , Sasaki T., Gilbert D.M. High-resolution Repli-Seq defines the temporal choreography of initiation, elongation and termination of replication in mammalian cells. Genome Biol. 2020; 21:76. 32209126 10.1186/s13059-020-01983-8 PMC7092589

[58] Huvet M. , Nicolay S., Touchon M., Audit B., Aubenton-Carafa Y., Arneodo A., Thermes C. Human gene organization driven by the coordination of replication and transcription. Genome Res. 2007; 17:1278–1285. 17675363 10.1101/gr.6533407 PMC1950896

[59] Hyrien O. , Rappailles A., Guilbaud G., Baker A., Chen C.L., Goldar A., Petryk N., Kahli M., Ma E., Aubenton-Carafa Y. et al. From simple bacterial and archaeal replicons to replication N/U-domains. J. Mol. Biol. 2013; 425:4673–4689. 24095859 10.1016/j.jmb.2013.09.021

