# Supplementary material for: DNA molecular combing-based replication fork directionality profiling
Source: Nucleic Acids Res. 2021 Apr 9;49(12):e69. doi: 10.1093/nar/gkab219 (PMC8266662; doi:10.1093/nar/gkab219)
Supplement: gkab219_Supplemental_File [file gkab219_supplemental_file.pdf]

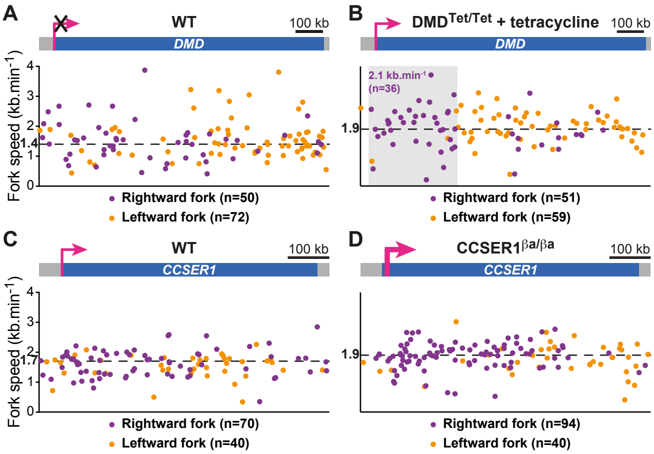

Supplementary Figure 1. Fork speed distribution along the *DMD* and *CCSER1* loci. (**A, B**) Fork speed distribution along *DMD* in WT cells (**A**) and $DMD^{Tet/Tet}$ cells with tetracycline (**B**) (data from Supplementary Figure 4A in (46), see Material and Methods). The speed of each individual replication fork overlapping *DMD* is plotted along the locus, represented as in Figure 2. Rightward- and leftward-moving forks are in purple and orange, respectively. The number of measurements (n) is indicated for each sample. Dotted lines represent median fork speeds, indicated on the left. In (**B**), the grey rectangle delimits the region of *DMD* replicated by rightward moving forks emanating from the IZ located 5' upstream of the gene in $DMD^{Tet/Tet}$ cells with tetracycline; the median velocity and number of these forks is indicated in the upper left corner. (**C, D**) Fork speed distribution along *CCSER1* in WT (**C**) and $CCSER1^{\beta a/\beta a}$ cells (**D**) (data from Supplementary Figure 4B in (46), see Material and Methods). In (**D**), the median speed of rightward forks emanating from the IZ in 5' of *CCSER1* and that of leftward forks coming from the IZ in 3' of the gene were 1.9 $kb.min^{-1}$ (n=90) and 1.8 $kb.min^{-1}$ (n=35), respectively; please note that forks undoubtedly originating from intragenic initiation events were excluded from median velocity calculations, which explains the slight difference with the total number of rightward (n=94) and leftward (n=40) forks.

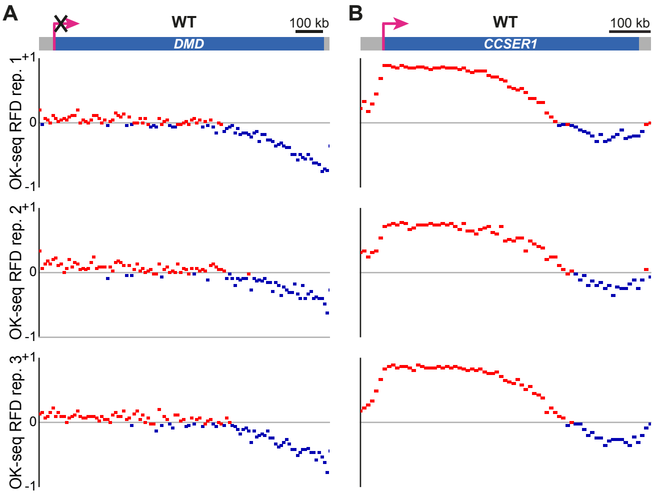

Supplementary Figure 2. OK-seq-based RFD profiles of WT *DMD* and *CCSER1* loci in three biological replicates. OK-seq data for WT *DMD* (**A**) and *CCSER1* (**B**) were binned into 10 kb adjacent windows. WT *DMD* and *CCSER1* loci are represented as in Figures 2 and 3, respectively. Rep., replicate.
